# Higher risk of knee arthroplasty during ten-year follow-up if baseline radiographic osteoarthritis involves the patellofemoral joint: a CHECK Cohort Study

**DOI:** 10.1186/s12891-022-05549-6

**Published:** 2022-06-22

**Authors:** Erin M. Macri, Marienke van Middelkoop, Jurgen Damen, P Koen Bos, Sita MA Bierma-Zeinstra

**Affiliations:** 1 Department of General Practice, Erasmus University Medical Center Rotterdam, Doctor Molewaterplein 40, 3015 GD Rotterdam, Netherlands; 2 Department of Orthopaedics and Sports Medicine, Erasmus University Medical Center Rotterdam, Doctor Molewaterplein 40, 3015 GD Rotterdam, Netherlands

**Keywords:** Patellofemoral joint, Tibiofemoral joint, Knee arthroplasty, Cohort study

## Abstract

**Background:**

Patellofemoral OA is a strong risk factor for progression to generalized whole knee OA, but it is unknown whether involvement of the patellofemoral joint in early radiographic OA (ROA) is associated with risk of undergoing future knee arthroplasty. This is clinically relevant because patellofemoral OA likely requires a different treatment approach than tibiofemoral OA, and identifying prognostic factors for future arthroplasty might assist clinicians with prioritizing and guiding early interventions that could improve long-term outcomes. Therefore, we evaluated association of baseline patellofemoral or tibiofemoral ROA with undergoing knee arthroplasty over 10 years.

**Methods:**

Using the multicenter Cohort Hip and Cohort Knee (CHECK) study, we acquired three views of radiographs in both knees of individuals aged 45–65 years with complaints of knee symptoms in at least one knee. From baseline radiographs, we categorized each knee as having one of four patterns of ROA: no ROA, isolated patellofemoral ROA, isolated tibiofemoral ROA, or combined ROA. We evaluated the 10-year relative hazard for undergoing going arthroplasty, based on baseline ROA pattern, using Cox proportional hazard models, adjusting for age, sex body mass index, and pain severity.

**Result:**

Our sample (*n* = 842) included 671 (80%) women and had mean (SD) age 56 (5) years, and BMI 26.3 (4.0) kg/m^2^. Arthroplasties were undertaken in 44/1678 knees. In comparison to having no ROA at baseline, adjusted hazard ratios (aHR) for arthroplasty were highest for combined ROA (aHR 14.2 [95% CI 5.8, 34.6]) and isolated patellofemoral ROA (aHR 12.7 [5.6, 29.0]). Isolated tibiofemoral ROA was not significantly associated with arthroplasty (aHR 2.9 [0.6, 13.6]).

**Conclusions:**

In a sample of middle-aged individuals with complaints in one or both knees, the 10-year relative hazard for undergoing arthroplasty, compared to no ROA, was increased when OA involved the patellofemoral joint, regardless of whether it was isolated to the patellofemoral joint or occurred in combination with tibiofemoral OA. Further research is needed to confirm this association and to clarify the causal mechanism of this relationship. However, our results provide preliminary evidence that identifying patellofemoral ROA may be a clinically useful prognostic indicator in early knee OA.

**Supplementary Information:**

The online version contains supplementary material available at 10.1186/s12891-022-05549-6.

## Background

Knee osteoarthritis (OA) is a chronic musculoskeletal condition associated with pain, loss of function and reduced quality of life [1]. Many individuals with knee OA experience progressive changes that, when severe enough, require extensive joint preserving strategies such as partial or total knee arthroplasty. Identifying important prognostic factors early in the disease trajectory could assist clinicians with prioritizing and guiding early interventions that have the potential to substantially improve long-term outcomes in knee OA. Knee OA affects both the tibiofemoral and patellofemoral joints, yet OA research has overwhelmingly focused on the tibiofemoral joint. Patellofemoral OA is a strong risk factor for progression to generalized whole knee OA [2–4]. It affects at least 25% of population-based cohorts [5–7], and it is associated with similar levels of pain, stiffness, loss of function and reduced quality of life as tibiofemoral OA [6, 8, 9]. What we do not know is whether the pattern of early radiographic OA (tibiofemoral, patellofemoral, or both combined) is prognostic of who will eventually require knee arthroplasty. This is particularly relevant since patellofemoral OA likely requires a different treatment approach than tibiofemoral OA [10–12]. To determine whether patellofemoral joint involvement in early OA is prognostic of clinical outcomes, we evaluated the relative hazard for undergoing arthroplasty according to baseline presence of patellofemoral or tibiofemoral OA (compared to no radiographic OA) in a cohort of middle-aged individuals with knee complaints.

## Methods

### Sample characteristics

The Cohort Hip and Cohort Knee (CHECK) study is a multicenter Dutch cohort of 1002 individuals aged 45–65 years at baseline who reported symptom complaints in the hip or knee. We evaluated a subgroup (*n* = 845) of individuals who reported knee pain or stiffness in one or both knees at baseline [2]. We defined symptom complaints as pain or stiffness for which the individual had never visited a physician for these complaints, or had first seen a physician for the symptoms less than six months prior to study enrolment, though their symptoms could have been present for longer than six months. Ethics approval was provided by all participating centers, and all participants provided informed written consent [13]. Research was carried out in accordance with the Helsinki Declaration.

### Radiographs and scoring

Radiographs of both knees were obtained at baseline, and were repeated during years two, five, eight and ten. Radiographs taken included: weightbearing posteroanterior (semi-flexed 7–10 $^\circ$), weightbearing lateral (flexed 30 $^\circ$) and non-weightbearing skyline (flexed 30 $^\circ$). Radiographs were scored for individual features using two atlases [14, 15], and Kellgren & Lawrence grades were assigned [16]. Radiographs across all time points from baseline to 10 years follow-up were read at the same time and readers were aware of their sequence in time [17]. Inter-rater reliability was previously established with prevalence and bias adjusted kappa of 0.6 [18].

We used all three views of baseline radiographs to define the OA pattern. We defined tibiofemoral compartment radiographic OA as Kellgren & Lawrence Grade ≥ 2 [16]. We defined patellofemoral radiographic OA as osteophytes of Grade ≥ 2, or joint space narrowing Grade ≥ 2 plus osteophytes Grade ≥ 1 [14]. Using these definitions, we categorized each knee as having one of four patterns of radiographic OA at baseline: no OA, isolated patellofemoral OA, isolated tibiofemoral OA, or combined OA. In secondary analyses, we evaluated individuals with any patellofemoral OA (regardless of tibiofemoral OA status), and also evaluated individuals with any tibiofemoral OA (regardless of patellofemoral OA status).

### Outcome

Our planned approach was to radiographically confirm the occurrence of arthroplasty (partial or total) over the 10 years of follow-up, and record the number of years from baseline to when the arthroplasty occurred. However, during our initial analyses, we identified seven cases where participants reported undergoing arthroplasty (including year the surgery took place) but we did not have radiographs to visually confirm those reports. Often it was the only joint with a missing radiograph (i.e., contralateral knee and bilateral hip images were still acquired). Thus, having an arthroplasty may have been the reason why radiographs were not taken (though this cannot be confirmed). We therefore defined the primary outcome as radiographically confirmed arthroplasty, but also considered cases of self-reported arthroplasty (that could not be confirmed radiographically) as a secondary outcome to account for possible misclassification of those cases.

### Statistical analyses

We evaluated relative hazard for undergoing going arthroplasty for all knees, based on baseline OA pattern, using Cox proportional hazard models, clustered at the participant level in order to account for the correlation between both knees within each participant. We defined no OA as our reference group. Individuals who withdrew from the study, were lost to follow-up, or did not undergo arthroplasty by the end of completion of the full study follow-up period were censored in the last year that data for a participant was recorded. In addition to crude hazard ratios (HR), we also adjusted for age, sex, body mass index (BMI), and baseline pain severity according to the Western Ontario McMaster Pain subscale (WOMAC Pain), which are known confounders related to both radiographic OA and future knee arthroplasty [19, 20]. After running each model, we performed proportional hazards tests and created log–log plots to confirm that assumptions were not violated.

In secondary analyses, we evaluated relative hazard for undergoing arthroplasty based on having any patellofemoral OA (regardless of tibiofemoral OA status) at baseline in comparison to having no radiographic patellofemoral OA. For these analyses, we estimated crude and adjusted HRs as above, and in a third model also adjusted for baseline tibiofemoral OA. Finally, we did the same evaluations for any baseline tibiofemoral OA, adjusting for baseline patellofemoral OA in the third model.

In addition to performing all of the above analyses with radiographically confirmed arthroplasties as our outcome, we performed sensitivity analyses based on the second definition of arthroplasty (either radiographic confirmation or self-reported) to consider the possibility of misclassification of several knees. Finally, we performed sensitivity analyses of radiographically confirmed arthroplasties in a subsample of knees with pain at study enrolment (i.e. excluding all asymptomatic knees from the analysis).

All statistical analyses were performed using Stata/SE 15.1 (StataCorp, Texas, US). We defined statistical significance as *p* < 0.05.

## Results

**Fig. 1:**
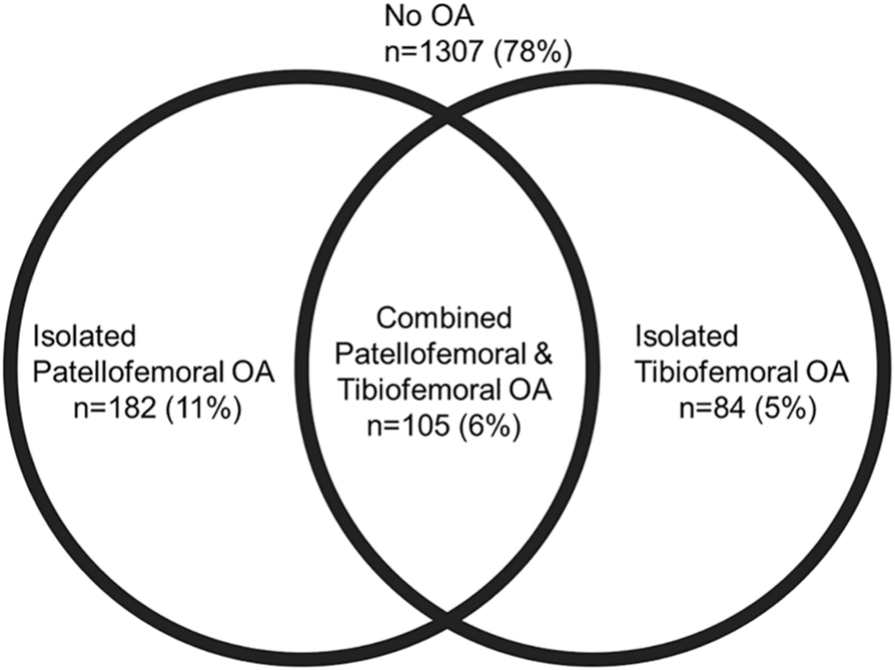
Proportions of knees by radiographic OA pattern (no OA, isolated patellofemoral OA, isolated tibiofemoral OA, combined patellofemoral and tibiofemoral OA)

Complete baseline radiographs were available and scored in 842 participants (1678 knees). This sample was comprised of 671 (80%) women, mean (SD) age 56 (5) years, and BMI 26.3 (4.0) kg/m^2^ (Table 1 reports these plus additional patient demographics). There was no radiographic OA in 1307 (78%) knees (Fig. 1, Table 2). Tibiofemoral OA was present in 189 (11%) knees, and was isolated to the tibiofemoral joint in 84 (5%) knees. Patellofemoral OA was present in 287 (17%) knees, and was isolated to the patellofemoral joint in 182 (11%) knees. Thus, combined tibiofemoral and patellofemoral OA was present in 105 (6%) knees.

**Table 1:** Participant baseline demographics

Characteristic	*N* = 842
Women, n (%)	671 (80%)
Age, mean (SD) years	55.9 (5.2)
BMI, mean (SD) m/kg^2^	26.3 (4.0)
Race, n (%)	
White	816 (97%)
Black	8 (1%)
Asian	12 (1%)
Other	5 (< 1%)
Comorbidities, n (%)	
None	212 (25%)
1–2	437 (52%)
3 +	177 (21%)
Kellgren & Lawrence Grade (*n* = 1684 knees)	
0	1010 (60%)
1	485 (29%)
2	187 (11%)
3	2 (< 1%)
4	0 (0%)
WOMAC, mean (SD) standardized scores/100	
Pain	25.6 (17.3)
Stiffness	33.7 (21.2)
Function	23.9 (17.3)
Duration of pain at enrollment, median (IQR) months (*n* = 729)	15 (9, 36)

*Note*: WOMAC scores and duration of pain are not joint-specific

*BMI* body mass index, *WOMAC* Western Ontario McMaster questionnaire, standardized to a scale from 0 to 100, with higher scores representing worse symptoms, *IQR* interquartile range

**Table 2:** Radiographically confirmed arthroplasties: hazard ratios (HR, 95% CI) for knees (*n* = 1678 due to missing data for 12 knees at baseline) undergoing arthroplasty over 10-years of follow-up, based on baseline OA compartment involvement (compared to no OA)

**Radiographic OA pattern**	**Number of arthroplasties**^a^ **/ baseline OA prevalence (%)**	**Crude HR (95% CI)**	**Adjusted HR (95% CI) **^b^
No OA	11 / 1307 (1%)	[ref]	[ref]
Isolated patellofemoral OA	17 / 182 (9%)	**11.9 (5.6, 25.4)**	**12.7 (5.6, 29.0)**
Isolated tibiofemoral OA	2 / 84 (2%)	2.9 (0.6, 13.3)	2.9 (0.6, 13.6)
Combined OA	13 / 105 (12%)	**16.6 (7.0, 39.1)**	**14.2 (5.8, 34.6)**
Any patellofemoral OA	30 / 287 (10%)	**12.2 (6.3, 23.6)**	**11.9 (5.8, 24.3)** **10.8 (4.9, 24.2)** ^c^
Any tibiofemoral OA	15 / 189 (8%)	**4.4 (2.2, 8.7)**	**3.7 (1.9, 7.4)** 1.3 (0.6, 2.8) ^c^

^a^ 43 arthroplasties included in analysis because 1 knee did not have complete baseline radiography

^b^ Adjusted for age, sex, body mass index and pain severity (WOMAC pain) at baseline

^c^ Additional adjustment by OA of the other compartment (i.e. add ‘any tibiofemoral OA’ to the patellofemoral OA model, and vice versa)

**Bold** indicates *p* < *0.05*

**Fig. 2:**
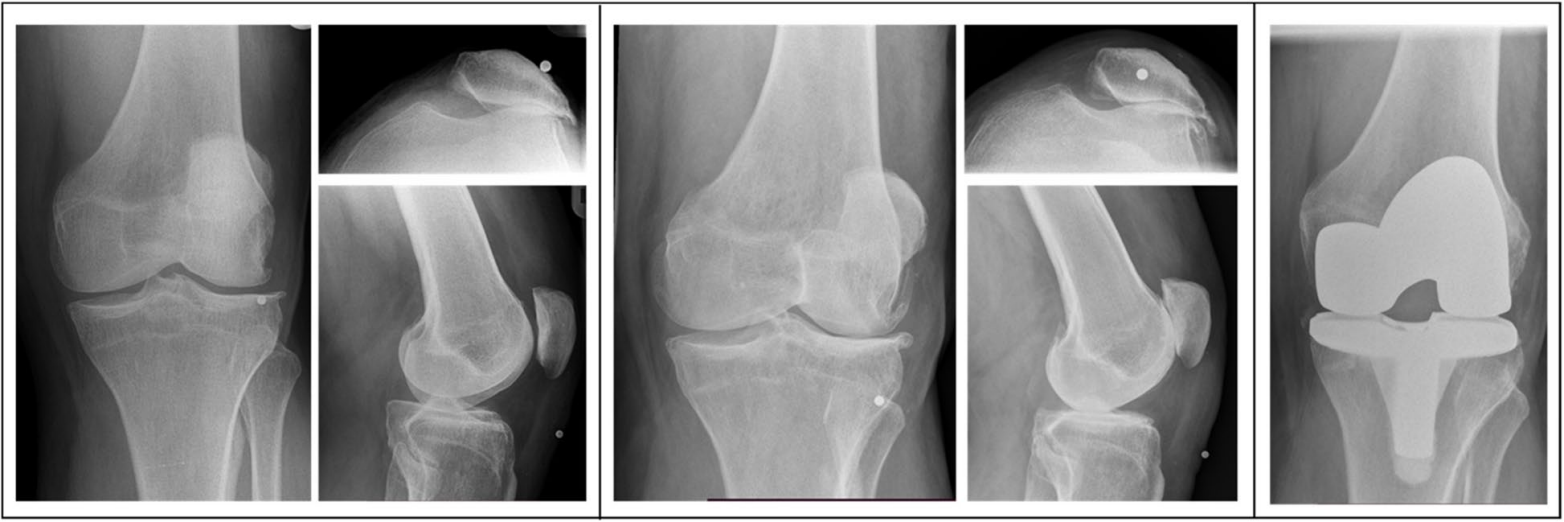
Participant with isolated patellofemoral OA (left panel, baseline) who progressed to combined OA (middle panel shows images at year 8), and finally underwent knee replacement (right panel, year 10)

Participant retention in the CHECK study was high, with 715 (85%) participants completing the 10-year follow-up; however, all 842 participants were included in our survival analyses. Arthroplasties were confirmed radiographically in 44 (3%) knees, 34 of which were total arthroplasties and 10 of which were medial hemi-arthroplasties (Fig. 2). Median time to arthroplasty for this group was 7 (interquartile range [IQR] 4) years: 8(3) for the subgroup with no baseline radiographic OA; 6(4) for isolated patellofemoral OA; 5.5(9) for isolated tibiofemoral OA; and 7(2) for combined OA. We also identified an additional seven arthroplasties that had been reported by participants but could not be radiographically confirmed (and were therefore of unknown type), thus 51 arthroplasties may have occurred. Median time to arthroplasty for this group was also 7 (IQR4) years: 8(4) for no baseline radiographic OA; 6(4) for isolated patellofemoral OA; 5.5(9) for isolated tibiofemoral OA; and 7.5(2) for combined OA. In comparison to having no OA at baseline, adjusted hazard ratios (HR) for radiographically confirmed arthroplasty were highest for combined OA (adjusted HR 14.2 [95% CI 5.8, 34.6]), followed by isolated patellofemoral OA (HR 12.7 [5.6, 29.0]) (Table 2). Isolated tibiofemoral OA was not significantly associated with arthroplasty (HR 2.9 [0.6, 13.6]).

When considering any patellofemoral OA (i.e., isolated or combined), associations were similar (adjusted HR 11.9 [5.8, 24.3]), and did not change substantially after adjusting for the presence of tibiofemoral OA (Table 2). When considering any tibiofemoral OA, the initially adjusted models were statistically significant (HR 3.7 [1.9, 7.4]), however further adjusting for presence of patellofemoral OA resulted in a smaller and non-significant association (HR 1.3 [0.6, 2.8]). Performing all analyses with the seven additional knees reclassified as having had an arthroplasty due to self-report (unable to confirm radiographically), results were similar, though with slightly attenuated effect sizes (Supplementary Table 1). Performing analyses with asymptomatic knees excluded, results again were similar, with slightly increased effect sizes and wider confidence intervals (Supplementary Table 2).

## Discussion

Our results suggest that in individuals with symptom complaints in one or both knees, those with patellofemoral joint involvement, whether isolated or in combination with tibiofemoral OA, were at higher risk of undergoing arthroplasty compared to those with no baseline OA. This finding should be interpreted within the context of the relatively low number of arthroplasties that took place in this cohort during the ten-year follow-up.

Because knee OA often begins in the patellofemoral joint and later progresses to whole knee OA, combined OA is generally believed to represent a more advanced stage of disease [4]. Thus, we expected that those at highest risk for undergoing arthroplasty would have combined OA. However, our results suggest that isolated patellofemoral OA may also be an important prognostic indicator regarding the risk for developing end-stage OA that requires arthroplasty.

Several possible theoretical frameworks could explain these findings, but they are preliminary at this stage. One possibility is that patellofemoral OA causes tibiofemoral OA which then leads to arthroplasty. This could occur through localized histochemical processes that communicate with and involve the tibiofemoral joint through shared synovium and intra-capsular space [1, 21]; or through biomechanical changes in response to pain, structural changes, or quadriceps weakness, resulting in increased mechanical loads to the tibiofemoral joint [21–28] that causes OA. A second possibility is that a distinct phenotype exists whereby OA begins in the whole knee but is only visualized in the patellofemoral joint in its earliest stages, thus patellofemoral OA is an early biomarker of whole-knee OA. This phenotype may represent a more severe form of OA that is at higher risk of arthroplasty than other phenotypes. A third possibility is that symptoms and loss of function are worse in knee OA if there is patellofemoral joint involvement [8], leading to a higher likelihood of a patient being selected for arthroplasty. Regardless of which explanation – if any—is correct, our results suggest that patellofemoral OA may be prognostic of clinical outcomes. Predicting, or forecasting, future events serves a clinically useful role independently of known causation [29]. In the CHECK cohort, of the 17 knees with isolated patellofemoral OA that went on to undergo arthroplasty, 12 knees progressed to combined OA prior to replacement. The remaining five knees may also have undergone progression to combined OA, but this may have been missed because of our imaging schedule (2 to 3 years between each follow-up visit).

Arthroplasty is not a ‘cure’ for OA, and approximately 20% who undergo knee arthroplasty report being unsatisfied following surgery [30, 31]. The findings of the present study bring up the important question of whether OA status at the patellofemoral joint is being adequately considered when making surgical treatment decisions, and whether this may be a factor that influences surgical decision making and outcomes. In the Netherlands, almost 80% of total knee arthroplasties are performed without resurfacing the patella [32], and international rates of patellar resurfacing range between 4 – 82% by country [33]. Recent meta-analyses and reviews suggest that patellar resurfacing is likely not cost effective in individuals without patellofemoral OA [34]. However, resurfacing may reduce revision rates and possibly also post-operative anterior knee pain, particularly if resurfacing is performed selectively [33–37]. While potential complications such as component loosening or patellar fracture must be considered, it may be that some individuals would benefit from patellar resurfacing during primary arthroplasty in order to specifically address patellofemoral joint-related symptoms. Future studies specifically evaluating pre-arthroplasty patellofemoral joint structural features and symptoms with post-operative outcomes are needed to investigate whether the patellofemoral joint is an important predictor of surgical outcomes, and whether selective patellar resurfacing could improve arthroplasty outcomes. Knee OA commonly first manifests in the patellofemoral joint, and patellofemoral OA is associated with as much pain, stiffness, loss of function and reduced quality of life as tibiofemoral OA [2–4, 6, 8]. The present study adds to the literature by demonstrating that patellofemoral OA may also increase risk of undergoing future knee arthroplasty. Thus, a growing body of literature suggests that the patellofemoral joint should be a high priority in knee OA research [38]. Identifying patellofemoral OA in its early stages and intervening appropriately may serve to alter the trajectory of knee OA, mitigate symptoms, and delay or prevent the need for future surgery. Conservative treatments such as exercises targeting the patellofemoral joint, taping, and bracing may improve pain, patellofemoral alignment, and OA-related structural features such as bone marrow lesions [10–12]. Clinical trials are urgently needed to optimize current treatment approaches, particularly treatments that can influence long-term outcomes.

### Limitations

A limitation of the present study is that we do not have peri-surgical details about the arthroplasties that took place in the CHECK cohort. Second, self-reported questionnaires regarding symptoms were completed for each individual, but not separately for each joint (i.e., both hips and both knees), so we were unable to conduct knee-specific analyses of pain and function. Third, individuals with meniscus or ligament injuries were excluded from the CHECK cohort, and since these are both risk factors for future knee arthroplasty [39], this may have led to an overestimation of our results and limits generalizability of our findings to a general knee OA population. It is also noted that our sample consisted of approximately 80% women. While we did adjust for sex in our model, it may be that the true associations between OA pattern and arthroplasty differ by sex. Finally, this study includes a relatively small number of individuals who underwent arthroplasty, thus effect sizes were imprecise and may not represent a stable point estimate. However, to our knowledge, the CHECK cohort is the largest early OA cohort that has adequate patellofemoral imaging and is therefore most suited to this research question. Other similar cohorts have not consistently obtained three views of radiographs, likely underestimating patellofemoral OA prevalence; or MRIs have only been acquired or scored in portions of study samples with limited follow-up or excluding individuals who do not undergo future arthroplasty [40–42]. Our results should be interpreted cautiously, however, they provide clinically relevant early epidemiological evidence that warrants future cohort studies with larger sample sizes and adequate patellofemoral imaging to confirm our findings.

## Conclusions

In a sample of middle-aged individuals with complaints of knee pain or stiffness in one or both knees, the relative hazard for undergoing arthroplasty within 10 years was substantially increased in those with radiographic patellofemoral OA, regardless of whether it was isolated to the patellofemoral joint or occurred in combination with tibiofemoral OA. Our results suggest that identifying patellofemoral joint involvement may serve as a clinically useful prognostic indicator in early knee OA.

## Supplementary Information

**Additional file 1: Supplementary Table S1. **Sensitivity analyses: Radiographically confirmed or self-reported arthroplasties: hazard ratios (HR, 95% CI) for knees (*n*=1678) undergoing knee arthroplasty over10-years of follow-up, based on baseline OA compartment involvement (compared to no OA).

**Additional file 2: Supplementary Table S2. **Sensitivity analyses: Radiographically confirmed arthroplasties in subset of knees with pain at enrolment: hazard ratios (HR, 95% CI) for knees (*n*=1281) undergoing knee arthroplasty over 10-years of follow-up, based on baseline OA compartment involvement (compared to no OA).

## Data Availability

The data generated during the CHECK study are archived in the publically available DANS (Data Archiving and Networking Services) Easy repository, https://easy.dans.knaw.nl/ui/datasets/id/easy-dataset:63523. Requests for collaboration can be sent to checkreu@umcutrecht.nl or m.wenting@umcutrecht.nl.

## Abbreviations

BMI: Body Mass Index
CHECK: Cohort Hip and Cohort Knee
CI: Confidence Interval
HR: Hazard Ratios
MRI: Magnetic Resonance Imaging
OA: Osteoarthritis
SD: Standard Deviation
WOMAC: Western Ontario McMaster Questionnaire
