# Supplementary figures and images for: Higher risk of knee arthroplasty during ten-year follow-up if baseline radiographic osteoarthritis involves the patellofemoral joint: a CHECK Cohort Study
Source: BMC Musculoskelet Disord. 2022 Jun 22;23:600. doi: 10.1186/s12891-022-05549-6 (PMC9215039; doi:10.1186/s12891-022-05549-6)

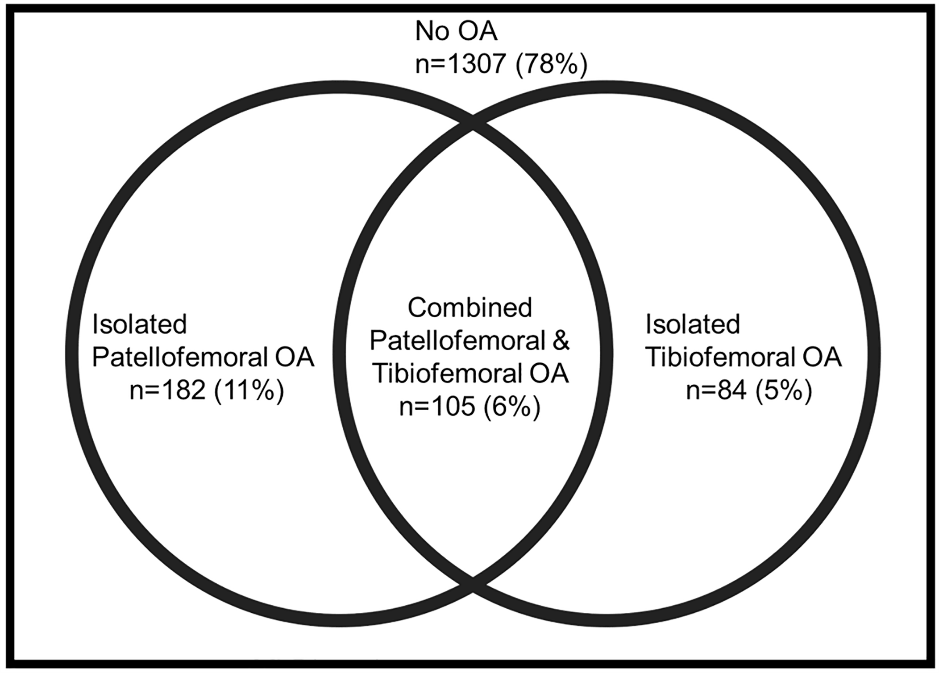

Supplement: Supplementary file 1 — Additional file 1: Supplementary Table S1. Sensitivity analyses: Radiographically confirmed or self-reported arthroplasties: hazard ratios (HR, 95% CI) for knees (n=1678) undergoing knee arthroplasty over10-years of follow-up, based on baseline OA compartment involvement (compared to no OA). [file 12891_2022_5549_MOESM1_ESM.jpeg]

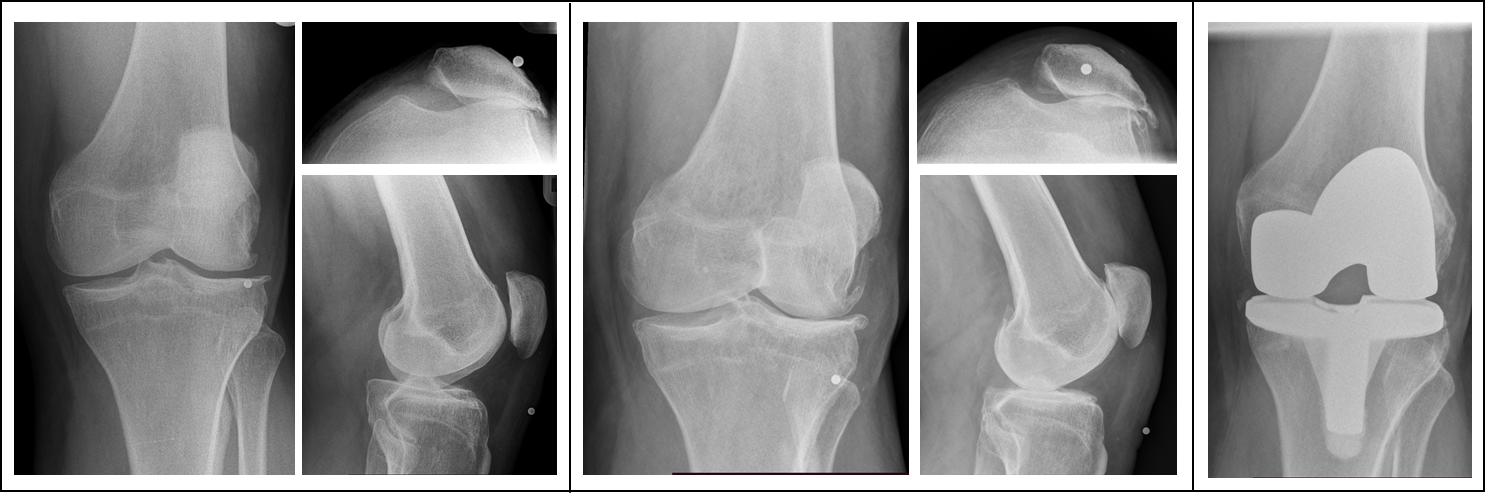

Supplement: Supplementary file 2 — Additional file 2: Supplementary Table S2. Sensitivity analyses: Radiographically confirmed arthroplasties in subset of knees with pain at enrolment: hazard ratios (HR, 95% CI) for knees (n=1281) undergoing knee arthroplasty over 10-years of follow-up, based on baseline OA compartment involvement (compared to no OA). [file 12891_2022_5549_MOESM2_ESM.png]
